# Supplementary material for: Path2Models: large-scale generation of computational models from biochemical pathway maps
Source: BMC Syst Biol. 2013 Nov 1;7:116. doi: 10.1186/1752-0509-7-116 (PMC4228421; doi:10.1186/1752-0509-7-116)
Supplement: Additional file 2 — Provided as an additional file and through labarchives, DOI:10.6070/H4WH2MX0. [file 1752-0509-7-116-S2.zip › Subliminal Toolbox v2/doc/mcisb-subliminal-lite/allclasses-frame.html]

All Classes


**All Classes**
  

|  |
| --- |
| BiomassExtracter   ChebiUtils   CobraFormatter   Extracter   FluxBoundsGenerater   GeneAssociationGenerater   KeggExtracter   KeggUtils   KeggUtilsTest   MetaCycExtracter   MetaCycUtils   MetaCycUtilsTest   ModelGenerater   MxnRefChemUtils   MxnRefChemUtilsTest   MxnRefReactionUtils   MxnRefReactionUtilsTest   MxnRefUtils   MxnRefUtils.Evidence   Path2ModelsReconstructionGenerator   SbmlFactory   SimpleMerger   SubliminalUtils   SubliminalUtilsTest   TransportExtracter   XmlFormatter |
