# Supplementary material for: Path2Models: large-scale generation of computational models from biochemical pathway maps
Source: BMC Syst Biol. 2013 Nov 1;7:116. doi: 10.1186/1752-0509-7-116 (PMC4228421; doi:10.1186/1752-0509-7-116)
Supplement: Additional file 2 — Provided as an additional file and through labarchives, DOI:10.6070/H4WH2MX0. [file 1752-0509-7-116-S2.zip › Subliminal Toolbox v2/doc/mcisb-subliminal-lite/constant-values.html]

Constant Field Values


---


|  |  |  |  |  |  |  |  |  |  |
| --- | --- | --- | --- | --- | --- | --- | --- | --- | --- |
| |  |  |  |  |  |  |  | | --- | --- | --- | --- | --- | --- | --- | | **Overview** | Package | Class | **Tree** | **Deprecated** | **Index** | **Help** | | |  |
| PREV   NEXT | **FRAMES**    **NO FRAMES**     **All Classes** |


---


# Constant Field Values


---

**Contents**

- org.mcisb.\*

| org.mcisb.\* |
| --- |

| org.mcisb.subliminal\_lite.Extracter | | |
| --- | --- | --- |
| `protected static final java.lang.String` | `BIOMASS_COMPARTMENT_ID` | `"bm"` |
| `protected static final java.lang.String` | `DEFAULT_COMPARTMENT_ID` | `"i"` |
| `protected static final java.lang.String` | `EXTRACELLULAR_COMPARTMENT_ID` | `"e"` |

| org.mcisb.subliminal\_lite.SubliminalUtils | | |
| --- | --- | --- |
| `public static final java.lang.String` | `BIOMASS_REACTION` | `"BIOMASS_REACTION"` |
| `public static final java.lang.String` | `CHARGE` | `"CHARGE"` |
| `public static final java.lang.String` | `COLON` | `":"` |
| `public static final double` | `DEFAULT_INITIAL_CONCENTRATION` | `1.0` |
| `public static final int` | `DEFAULT_LEVEL` | `2` |
| `public static final int` | `DEFAULT_VERSION` | `4` |
| `public static final java.lang.String` | `EMPTY_STRING` | `""` |
| `public static final int` | `FIRST` | `0` |
| `public static final java.lang.String` | `FORMULA` | `"FORMULA"` |
| `public static final java.lang.String` | `HYPHEN` | `"-"` |
| `public static final java.lang.String` | `INCHI` | `"INCHI"` |
| `public static final java.lang.String` | `NON_WORD` | `"\\W+"` |
| `public static final int` | `SBO_BIOCHEMICAL_REACTION` | `176` |
| `public static final int` | `SBO_COMPARTMENT` | `290` |
| `public static final int` | `SBO_OMITTED_PROCESS` | `397` |
| `public static final int` | `SBO_POLYPEPTIDE_CHAIN` | `252` |
| `public static final int` | `SBO_PROTEIN_COMPLEX` | `297` |
| `public static final int` | `SBO_SIMPLE_CHEMICAL` | `247` |
| `public static final int` | `SBO_TRANSPORT_REACTION` | `185` |
| `public static final java.lang.String` | `SMILES` | `"SMILES"` |
| `public static final int` | `UNDEFINED_NUMBER` | `-2147483648` |
| `public static final java.lang.String` | `UNDERSCORE` | `"_"` |
| `public static final java.lang.String` | `WHITESPACE` | `"\\s+"` |

---


|  |  |  |  |  |  |  |  |  |  |
| --- | --- | --- | --- | --- | --- | --- | --- | --- | --- |
| |  |  |  |  |  |  |  | | --- | --- | --- | --- | --- | --- | --- | | **Overview** | Package | Class | **Tree** | **Deprecated** | **Index** | **Help** | | |  |
| PREV   NEXT | **FRAMES**    **NO FRAMES**     **All Classes** |


---
