# Supplementary material for: Path2Models: large-scale generation of computational models from biochemical pathway maps
Source: BMC Syst Biol. 2013 Nov 1;7:116. doi: 10.1186/1752-0509-7-116 (PMC4228421; doi:10.1186/1752-0509-7-116)
Supplement: Additional file 2 — Provided as an additional file and through labarchives, DOI:10.6070/H4WH2MX0. [file 1752-0509-7-116-S2.zip › Subliminal Toolbox v2/doc/mcisb-subliminal-lite/index-all.html]

Index


---


|  |  |  |  |  |  |  |  |  |  |
| --- | --- | --- | --- | --- | --- | --- | --- | --- | --- |
| |  |  |  |  |  |  |  | | --- | --- | --- | --- | --- | --- | --- | | **Overview** | Package | Class | **Tree** | **Deprecated** | **Index** | **Help** | | |  |
| PREV   NEXT | **FRAMES**    **NO FRAMES**     **All Classes** |


A B C D E F G H I K M N O P R S T U V W X 

---


## **A**

**addCompartment(Model, String, String)** - Static method in class org.mcisb.subliminal\_lite.Extracter: **addCVTerm(SBase, String, CVTerm.Type, CVTerm.Qualifier)** - Static method in class org.mcisb.subliminal\_lite.SubliminalUtils: **addCVTerms(SBase, Collection<String>, CVTerm.Type, CVTerm.Qualifier)** - Static method in class org.mcisb.subliminal\_lite.SubliminalUtils: **addDefaultCompartment(Model)** - Static method in class org.mcisb.subliminal\_lite.Extracter: **addEnzyme(Reaction, String, String, Collection<Object[]>)** - Static method in class org.mcisb.subliminal\_lite.Extracter: **addEnzymes(Reaction, List<String[]>, String, String, Collection<Object[]>)** - Static method in class org.mcisb.subliminal\_lite.Extracter: **addHistory(SBMLDocument)** - Static method in class org.mcisb.subliminal\_lite.SubliminalUtils: **addNote(SBase, String, Object)** - Static method in class org.mcisb.subliminal\_lite.SubliminalUtils: **addReaction(Model, String, String)** - Static method in class org.mcisb.subliminal\_lite.Extracter: **addResources(SBase, Collection<Object[]>)** - Static method in class org.mcisb.subliminal\_lite.Extracter: **addSpecies(Model, String, String, String, int)** - Static method in class org.mcisb.subliminal\_lite.Extracter: **addSpecies(Model, String, String, String, int, Collection<Object[]>)** - Static method in class org.mcisb.subliminal\_lite.Extracter

---


## **B**

**BIOMASS\_COMPARTMENT\_ID** - Static variable in class org.mcisb.subliminal\_lite.Extracter: **BIOMASS\_REACTION** - Static variable in class org.mcisb.subliminal\_lite.SubliminalUtils: **BiomassExtracter** - Class in org.mcisb.subliminal\_lite.model: **BiomassExtracter()** - Constructor for class org.mcisb.subliminal\_lite.model.BiomassExtracter

---


## **C**

**CHARGE** - Static variable in class org.mcisb.subliminal\_lite.SubliminalUtils: **ChebiUtils** - Class in org.mcisb.subliminal\_lite.xref: **ChebiUtils()** - Constructor for class org.mcisb.subliminal\_lite.xref.ChebiUtils: **CobraFormatter** - Class in org.mcisb.subliminal\_lite.model: **CobraFormatter()** - Constructor for class org.mcisb.subliminal\_lite.model.CobraFormatter: **COLON** - Static variable in class org.mcisb.subliminal\_lite.SubliminalUtils

---


## **D**

**DEFAULT\_COMPARTMENT\_ID** - Static variable in class org.mcisb.subliminal\_lite.Extracter: **DEFAULT\_INITIAL\_CONCENTRATION** - Static variable in class org.mcisb.subliminal\_lite.SubliminalUtils: **DEFAULT\_LEVEL** - Static variable in class org.mcisb.subliminal\_lite.SubliminalUtils: **DEFAULT\_VERSION** - Static variable in class org.mcisb.subliminal\_lite.SubliminalUtils: **delete(File)** - Static method in class org.mcisb.subliminal\_lite.SubliminalUtils

---


## **E**

**EMPTY\_STRING** - Static variable in class org.mcisb.subliminal\_lite.SubliminalUtils: **encodeUniProtSearchTerm(String)** - Static method in class org.mcisb.subliminal\_lite.SubliminalUtils: **EXTRACELLULAR\_COMPARTMENT\_ID** - Static variable in class org.mcisb.subliminal\_lite.Extracter: **Extracter** - Class in org.mcisb.subliminal\_lite: **Extracter()** - Constructor for class org.mcisb.subliminal\_lite.Extracter

---


## **F**

**find(File, String)** - Static method in class org.mcisb.subliminal\_lite.SubliminalUtils: **FIRST** - Static variable in class org.mcisb.subliminal\_lite.SubliminalUtils: **FluxBoundsGenerater** - Class in org.mcisb.subliminal\_lite.model: **FluxBoundsGenerater()** - Constructor for class org.mcisb.subliminal\_lite.model.FluxBoundsGenerater: **FORMULA** - Static variable in class org.mcisb.subliminal\_lite.SubliminalUtils

---


## **G**

**GeneAssociationGenerater** - Class in org.mcisb.subliminal\_lite.model: **GeneAssociationGenerater()** - Constructor for class org.mcisb.subliminal\_lite.model.GeneAssociationGenerater: **getBalance(String)** - Method in class org.mcisb.subliminal\_lite.mnxref.MxnRefReactionUtils: **getCharge(String)** - Method in class org.mcisb.subliminal\_lite.mnxref.MxnRefChemUtils: **getCompartmentalisedId(String, String)** - Static method in class org.mcisb.subliminal\_lite.SubliminalUtils: **getData(String, int, URL)** - Method in class org.mcisb.subliminal\_lite.mnxref.MxnRefUtils: **getDecompartmentalisedId(String, String)** - Static method in class org.mcisb.subliminal\_lite.SubliminalUtils: **getDescription(String)** - Method in class org.mcisb.subliminal\_lite.mnxref.MxnRefReactionUtils: **getEC(String)** - Method in class org.mcisb.subliminal\_lite.mnxref.MxnRefReactionUtils: **getEquation(String)** - Method in class org.mcisb.subliminal\_lite.mnxref.MxnRefReactionUtils: **getEvidence()** - Method in class org.mcisb.subliminal\_lite.mnxref.MxnRefChemUtilsTest: **getEvidence(String)** - Method in class org.mcisb.subliminal\_lite.mnxref.MxnRefUtils: **getFormula(String)** - Method in class org.mcisb.subliminal\_lite.mnxref.MxnRefChemUtils: **getInchi(String)** - Method in class org.mcisb.subliminal\_lite.mnxref.MxnRefChemUtils: **getInchi()** - Method in class org.mcisb.subliminal\_lite.mnxref.MxnRefChemUtilsTest: **getInstance()** - Static method in class org.mcisb.subliminal\_lite.mnxref.MxnRefChemUtils: **getInstance()** - Static method in class org.mcisb.subliminal\_lite.mnxref.MxnRefReactionUtils: **getInstance()** - Static method in class org.mcisb.subliminal\_lite.model.XmlFormatter: **getInstance()** - Static method in class org.mcisb.subliminal\_lite.sbml.SbmlFactory: **getMxnRefId(String)** - Method in class org.mcisb.subliminal\_lite.mnxref.MxnRefUtils: **getName(String)** - Method in class org.mcisb.subliminal\_lite.mnxref.MxnRefChemUtils: **getName()** - Method in class org.mcisb.subliminal\_lite.mnxref.MxnRefChemUtilsTest: **getName()** - Method in class org.mcisb.subliminal\_lite.mnxref.MxnRefReactionUtilsTest: **getNormalisedId(String)** - Static method in class org.mcisb.subliminal\_lite.SubliminalUtils: **getNotes(SBase)** - Static method in class org.mcisb.subliminal\_lite.SubliminalUtils: **getNoteValues(SBase, String)** - Static method in class org.mcisb.subliminal\_lite.SubliminalUtils: **getOrganismIds()** - Static method in class org.mcisb.subliminal\_lite.kegg.KeggUtils: **getOrganismIds()** - Method in class org.mcisb.subliminal\_lite.kegg.KeggUtilsTest: **getOrganismName(String)** - Static method in class org.mcisb.subliminal\_lite.kegg.KeggUtils: **getReaction(String, String)** - Method in class org.mcisb.subliminal\_lite.sbml.SbmlFactory: **getSmiles(String)** - Method in class org.mcisb.subliminal\_lite.mnxref.MxnRefChemUtils: **getSpecies(String, String)** - Method in class org.mcisb.subliminal\_lite.sbml.SbmlFactory: **getTaxonomyId(String)** - Static method in class org.mcisb.subliminal\_lite.kegg.KeggUtils: **getTaxonomyName(String)** - Static method in class org.mcisb.subliminal\_lite.SubliminalUtils: **getUniqueId()** - Static method in class org.mcisb.subliminal\_lite.SubliminalUtils: **getXrefIds(String)** - Method in class org.mcisb.subliminal\_lite.mnxref.MxnRefUtils: **getXrefIds(String, String)** - Method in class org.mcisb.subliminal\_lite.mnxref.MxnRefUtils

---


## **H**

**HYPHEN** - Static variable in class org.mcisb.subliminal\_lite.SubliminalUtils

---


## **I**

**INCHI** - Static variable in class org.mcisb.subliminal\_lite.SubliminalUtils: **initDocument(String)** - Static method in class org.mcisb.subliminal\_lite.Extracter: **initXrefs()** - Method in class org.mcisb.subliminal\_lite.mnxref.MxnRefUtils: **isPrimary(String)** - Static method in class org.mcisb.subliminal\_lite.xref.ChebiUtils

---


## **K**

**KeggExtracter** - Class in org.mcisb.subliminal\_lite.kegg: **KeggExtracter()** - Constructor for class org.mcisb.subliminal\_lite.kegg.KeggExtracter: **KeggUtils** - Class in org.mcisb.subliminal\_lite.kegg: **KeggUtils()** - Constructor for class org.mcisb.subliminal\_lite.kegg.KeggUtils: **KeggUtilsTest** - Class in org.mcisb.subliminal\_lite.kegg: **KeggUtilsTest()** - Constructor for class org.mcisb.subliminal\_lite.kegg.KeggUtilsTest

---


## **M**

**main(String[])** - Static method in class org.mcisb.subliminal\_lite.kegg.KeggExtracter: **main(String[])** - Static method in class org.mcisb.subliminal\_lite.metacyc.MetaCycExtracter: **main(String[])** - Static method in class org.mcisb.subliminal\_lite.model.XmlFormatter: **main(String[])** - Static method in class org.mcisb.subliminal\_lite.Path2ModelsReconstructionGenerator: **MetaCycExtracter** - Class in org.mcisb.subliminal\_lite.metacyc: **MetaCycExtracter()** - Constructor for class org.mcisb.subliminal\_lite.metacyc.MetaCycExtracter: **MetaCycUtils** - Class in org.mcisb.subliminal\_lite.metacyc: **MetaCycUtils()** - Constructor for class org.mcisb.subliminal\_lite.metacyc.MetaCycUtils: **MetaCycUtilsTest** - Class in org.mcisb.subliminal\_lite.metacyc: **MetaCycUtilsTest()** - Constructor for class org.mcisb.subliminal\_lite.metacyc.MetaCycUtilsTest: **ModelGenerater** - Class in org.mcisb.subliminal\_lite.model: **ModelGenerater()** - Constructor for class org.mcisb.subliminal\_lite.model.ModelGenerater: **MxnRefChemUtils** - Class in org.mcisb.subliminal\_lite.mnxref: **MxnRefChemUtilsTest** - Class in org.mcisb.subliminal\_lite.mnxref: **MxnRefChemUtilsTest()** - Constructor for class org.mcisb.subliminal\_lite.mnxref.MxnRefChemUtilsTest: **mxnRefIdToXrefIds** - Variable in class org.mcisb.subliminal\_lite.mnxref.MxnRefUtils: **MxnRefReactionUtils** - Class in org.mcisb.subliminal\_lite.mnxref: **MxnRefReactionUtilsTest** - Class in org.mcisb.subliminal\_lite.mnxref: **MxnRefReactionUtilsTest()** - Constructor for class org.mcisb.subliminal\_lite.mnxref.MxnRefReactionUtilsTest: **MxnRefUtils** - Class in org.mcisb.subliminal\_lite.mnxref: **MxnRefUtils(String, URL)** - Constructor for class org.mcisb.subliminal\_lite.mnxref.MxnRefUtils: **MxnRefUtils.Evidence** - Enum in org.mcisb.subliminal\_lite.mnxref

---


## **N**

**NON\_WORD** - Static variable in class org.mcisb.subliminal\_lite.SubliminalUtils

---


## **O**

**org.mcisb.subliminal\_lite** - package org.mcisb.subliminal\_lite: **org.mcisb.subliminal\_lite.kegg** - package org.mcisb.subliminal\_lite.kegg: **org.mcisb.subliminal\_lite.merge** - package org.mcisb.subliminal\_lite.merge: **org.mcisb.subliminal\_lite.metacyc** - package org.mcisb.subliminal\_lite.metacyc: **org.mcisb.subliminal\_lite.mnxref** - package org.mcisb.subliminal\_lite.mnxref: **org.mcisb.subliminal\_lite.model** - package org.mcisb.subliminal\_lite.model: **org.mcisb.subliminal\_lite.sbml** - package org.mcisb.subliminal\_lite.sbml: **org.mcisb.subliminal\_lite.xref** - package org.mcisb.subliminal\_lite.xref

---


## **P**

**parseStoichiometry(String)** - Static method in class org.mcisb.subliminal\_lite.SubliminalUtils: **Path2ModelsReconstructionGenerator** - Class in org.mcisb.subliminal\_lite: **Path2ModelsReconstructionGenerator()** - Constructor for class org.mcisb.subliminal\_lite.Path2ModelsReconstructionGenerator

---


## **R**

**reconstruct(File, String)** - Static method in class org.mcisb.subliminal\_lite.Path2ModelsReconstructionGenerator: **reconstructAll(File)** - Static method in class org.mcisb.subliminal\_lite.Path2ModelsReconstructionGenerator: **reconstructFrom(File, String)** - Static method in class org.mcisb.subliminal\_lite.Path2ModelsReconstructionGenerator: **reconstructList(File, String[])** - Static method in class org.mcisb.subliminal\_lite.Path2ModelsReconstructionGenerator: **run(String, File)** - Static method in class org.mcisb.subliminal\_lite.kegg.KeggExtracter: **run(String)** - Static method in class org.mcisb.subliminal\_lite.kegg.KeggExtracter: **run(File[], File)** - Static method in class org.mcisb.subliminal\_lite.merge.SimpleMerger: **run(String, File)** - Static method in class org.mcisb.subliminal\_lite.metacyc.MetaCycExtracter: **run(String, SBMLDocument)** - Static method in class org.mcisb.subliminal\_lite.metacyc.MetaCycExtracter: **run(SBMLDocument)** - Static method in class org.mcisb.subliminal\_lite.model.BiomassExtracter: **run(SBMLDocument)** - Static method in class org.mcisb.subliminal\_lite.model.CobraFormatter: **run(SBMLDocument)** - Static method in class org.mcisb.subliminal\_lite.model.FluxBoundsGenerater: **run(SBMLDocument)** - Static method in class org.mcisb.subliminal\_lite.model.GeneAssociationGenerater: **run(File, File)** - Static method in class org.mcisb.subliminal\_lite.model.ModelGenerater: **run(SBMLDocument)** - Static method in class org.mcisb.subliminal\_lite.model.ModelGenerater: **run(SBMLDocument)** - Static method in class org.mcisb.subliminal\_lite.model.TransportExtracter

---


## **S**

**SbmlFactory** - Class in org.mcisb.subliminal\_lite.sbml: **SBO\_BIOCHEMICAL\_REACTION** - Static variable in class org.mcisb.subliminal\_lite.SubliminalUtils: **SBO\_COMPARTMENT** - Static variable in class org.mcisb.subliminal\_lite.SubliminalUtils: **SBO\_OMITTED\_PROCESS** - Static variable in class org.mcisb.subliminal\_lite.SubliminalUtils: **SBO\_POLYPEPTIDE\_CHAIN** - Static variable in class org.mcisb.subliminal\_lite.SubliminalUtils: **SBO\_PROTEIN\_COMPLEX** - Static variable in class org.mcisb.subliminal\_lite.SubliminalUtils: **SBO\_SIMPLE\_CHEMICAL** - Static variable in class org.mcisb.subliminal\_lite.SubliminalUtils: **SBO\_TRANSPORT\_REACTION** - Static variable in class org.mcisb.subliminal\_lite.SubliminalUtils: **searchUniProt(String)** - Static method in class org.mcisb.subliminal\_lite.SubliminalUtils: **searchUniProt()** - Method in class org.mcisb.subliminal\_lite.SubliminalUtilsTest: **setNotes(SBase, Map<String, Object>)** - Static method in class org.mcisb.subliminal\_lite.SubliminalUtils: **SimpleMerger** - Class in org.mcisb.subliminal\_lite.merge: **SimpleMerger()** - Constructor for class org.mcisb.subliminal\_lite.merge.SimpleMerger: **SMILES** - Static variable in class org.mcisb.subliminal\_lite.SubliminalUtils: **stripTags(String)** - Static method in class org.mcisb.subliminal\_lite.SubliminalUtils: **SubliminalUtils** - Class in org.mcisb.subliminal\_lite: **SubliminalUtils()** - Constructor for class org.mcisb.subliminal\_lite.SubliminalUtils: **SubliminalUtilsTest** - Class in org.mcisb.subliminal\_lite: **SubliminalUtilsTest()** - Constructor for class org.mcisb.subliminal\_lite.SubliminalUtilsTest

---


## **T**

**toString(Collection<?>)** - Static method in class org.mcisb.subliminal\_lite.SubliminalUtils: **TransportExtracter** - Class in org.mcisb.subliminal\_lite.model: **TransportExtracter()** - Constructor for class org.mcisb.subliminal\_lite.model.TransportExtracter

---


## **U**

**UNDEFINED\_NUMBER** - Static variable in class org.mcisb.subliminal\_lite.SubliminalUtils: **UNDERSCORE** - Static variable in class org.mcisb.subliminal\_lite.SubliminalUtils: **unencode(String)** - Static method in class org.mcisb.subliminal\_lite.metacyc.MetaCycUtils: **unencode()** - Method in class org.mcisb.subliminal\_lite.metacyc.MetaCycUtilsTest: **unregister()** - Method in class org.mcisb.subliminal\_lite.sbml.SbmlFactory: **untar(URL, File)** - Static method in class org.mcisb.subliminal\_lite.SubliminalUtils

---


## **V**

**valueOf(String)** - Static method in enum org.mcisb.subliminal\_lite.mnxref.MxnRefUtils.Evidence: Returns the enum constant of this type with the specified name. **values()** - Static method in enum org.mcisb.subliminal\_lite.mnxref.MxnRefUtils.Evidence: Returns an array containing the constants of this enum type, in the order they are declared.

---


## **W**

**WHITESPACE** - Static variable in class org.mcisb.subliminal\_lite.SubliminalUtils: **write(SBMLDocument, File)** - Method in class org.mcisb.subliminal\_lite.model.XmlFormatter: **write(File, File)** - Method in class org.mcisb.subliminal\_lite.model.XmlFormatter

---


## **X**

**XmlFormatter** - Class in org.mcisb.subliminal\_lite.model

---

A B C D E F G H I K M N O P R S T U V W X


|  |  |  |  |  |  |  |  |  |  |
| --- | --- | --- | --- | --- | --- | --- | --- | --- | --- |
| |  |  |  |  |  |  |  | | --- | --- | --- | --- | --- | --- | --- | | **Overview** | Package | Class | **Tree** | **Deprecated** | **Index** | **Help** | | |  |
| PREV   NEXT | **FRAMES**    **NO FRAMES**     **All Classes** |


---
