# Supplementary material for: Path2Models: large-scale generation of computational models from biochemical pathway maps
Source: BMC Syst Biol. 2013 Nov 1;7:116. doi: 10.1186/1752-0509-7-116 (PMC4228421; doi:10.1186/1752-0509-7-116)
Supplement: Additional file 2 — Provided as an additional file and through labarchives, DOI:10.6070/H4WH2MX0. [file 1752-0509-7-116-S2.zip › Subliminal Toolbox v2/doc/mcisb-subliminal-lite/org/mcisb/subliminal_lite/Extracter.html]

Extracter


---


|  |  |  |  |  |  |  |  |  |  |
| --- | --- | --- | --- | --- | --- | --- | --- | --- | --- |
| |  |  |  |  |  |  |  | | --- | --- | --- | --- | --- | --- | --- | | **Overview** | **Package** | **Class** | **Tree** | **Deprecated** | **Index** | **Help** | | |  |
| PREV CLASS   **NEXT CLASS** | **FRAMES**    **NO FRAMES**     **All Classes** |
| SUMMARY: NESTED | FIELD | CONSTR | METHOD | DETAIL: FIELD | CONSTR | METHOD |


---


## org.mcisb.subliminal\_lite Class Extracter

```
java.lang.Object
  org.mcisb.subliminal_lite.Extracter
```

**Direct Known Subclasses:**: BiomassExtracter, CobraFormatter, KeggExtracter, MetaCycExtracter, TransportExtracter

---

``` public abstract class Extracter extends java.lang.Object ```

**Author:**
:   Neil Swainston

---

| **Field Summary** | |
| --- | --- |
| `protected static java.lang.String` | `BIOMASS_COMPARTMENT_ID` |
| `protected static java.lang.String` | `DEFAULT_COMPARTMENT_ID` |
| `protected static java.lang.String` | `EXTRACELLULAR_COMPARTMENT_ID` |


| **Constructor Summary** | |
| --- | --- |
| `Extracter()` |


| **Method Summary** | |
| --- | --- |
| `protected static org.sbml.jsbml.Compartment` | `addCompartment(org.sbml.jsbml.Model model, java.lang.String id, java.lang.String name)` |
| `protected static void` | `addDefaultCompartment(org.sbml.jsbml.Model model)` |
| `protected static void` | `addEnzyme(org.sbml.jsbml.Reaction reaction, java.lang.String id, java.lang.String name, java.util.Collection<java.lang.Object[]> resources)` |
| `protected static void` | `addEnzymes(org.sbml.jsbml.Reaction reaction, java.util.List<java.lang.String[]> uniProtResults, java.lang.String geneId, java.lang.String geneName, java.util.Collection<java.lang.Object[]> resources)` |
| `protected static org.sbml.jsbml.Reaction` | `addReaction(org.sbml.jsbml.Model model, java.lang.String reactionId, java.lang.String compartmentId)` |
| `protected static void` | `addResources(org.sbml.jsbml.SBase sbase, java.util.Collection<java.lang.Object[]> resources)` |
| `protected static org.sbml.jsbml.Species` | `addSpecies(org.sbml.jsbml.Model model, java.lang.String speciesId, java.lang.String name, java.lang.String compartmentId, int sboTerm)` |
| `protected static org.sbml.jsbml.Species` | `addSpecies(org.sbml.jsbml.Model model, java.lang.String speciesId, java.lang.String name, java.lang.String compartmentId, int sboTerm, java.util.Collection<java.lang.Object[]> resources)` |
| `protected static org.sbml.jsbml.SBMLDocument` | `initDocument(java.lang.String taxonomyId)` |

| **Methods inherited from class java.lang.Object** |
| --- |
| `clone, equals, finalize, getClass, hashCode, notify, notifyAll, toString, wait, wait, wait` |

| **Field Detail** |
| --- |

### DEFAULT\_COMPARTMENT\_ID

```
protected static final java.lang.String DEFAULT_COMPARTMENT_ID
```

**See Also:**: Constant Field Values

---


### EXTRACELLULAR\_COMPARTMENT\_ID

```
protected static final java.lang.String EXTRACELLULAR_COMPARTMENT_ID
```

**See Also:**: Constant Field Values

---


### BIOMASS\_COMPARTMENT\_ID

```
protected static final java.lang.String BIOMASS_COMPARTMENT_ID
```

**See Also:**: Constant Field Values


| **Constructor Detail** |
| --- |

### Extracter

```
public Extracter()
```


| **Method Detail** |
| --- |

### initDocument

```
protected static org.sbml.jsbml.SBMLDocument initDocument(java.lang.String taxonomyId)
                                                   throws java.io.IOException
```

:   **Parameters:**: `taxonomyId` - **Returns:**: SBMLDocument **Throws:**: `java.io.IOException`

---


### addDefaultCompartment

```
protected static void addDefaultCompartment(org.sbml.jsbml.Model model)
```

:   **Parameters:**: `model` -

---


### addCompartment

```
protected static org.sbml.jsbml.Compartment addCompartment(org.sbml.jsbml.Model model,
                                                           java.lang.String id,
                                                           java.lang.String name)
```

:   **Parameters:**: `model` -: `id` -: `name` - **Returns:**: Compartment

---


### addSpecies

```
protected static org.sbml.jsbml.Species addSpecies(org.sbml.jsbml.Model model,
                                                   java.lang.String speciesId,
                                                   java.lang.String name,
                                                   java.lang.String compartmentId,
                                                   int sboTerm)
                                            throws java.lang.Exception
```

:   **Parameters:**: `model` -: `speciesId` -: `name` -: `compartmentId` -: `sboTerm` - **Returns:**: Species **Throws:**: `java.lang.Exception`

---


### addSpecies

```
protected static org.sbml.jsbml.Species addSpecies(org.sbml.jsbml.Model model,
                                                   java.lang.String speciesId,
                                                   java.lang.String name,
                                                   java.lang.String compartmentId,
                                                   int sboTerm,
                                                   java.util.Collection<java.lang.Object[]> resources)
                                            throws java.lang.Exception
```

:   **Parameters:**: `model` -: `speciesId` -: `name` -: `sboTerm` -: `resources` - **Returns:**: Species **Throws:**: `java.lang.Exception`

---


### addReaction

```
protected static org.sbml.jsbml.Reaction addReaction(org.sbml.jsbml.Model model,
                                                     java.lang.String reactionId,
                                                     java.lang.String compartmentId)
                                              throws java.lang.Exception
```

:   **Parameters:**: `model` -: `reactionId` - **Returns:**: Reaction **Throws:**: `java.lang.Exception`

---


### addEnzymes

```
protected static void addEnzymes(org.sbml.jsbml.Reaction reaction,
                                 java.util.List<java.lang.String[]> uniProtResults,
                                 java.lang.String geneId,
                                 java.lang.String geneName,
                                 java.util.Collection<java.lang.Object[]> resources)
                          throws java.lang.Exception
```

:   **Parameters:**: `reaction` -: `uniProtResults` -: `geneId` -: `geneName` -: `resources` - **Throws:**: `java.lang.Exception`

---


### addEnzyme

```
protected static void addEnzyme(org.sbml.jsbml.Reaction reaction,
                                java.lang.String id,
                                java.lang.String name,
                                java.util.Collection<java.lang.Object[]> resources)
                         throws java.lang.Exception
```

:   **Parameters:**: `reaction` -: `id` -: `name` -: `resources` - **Throws:**: `java.lang.Exception`

---


### addResources

```
protected static void addResources(org.sbml.jsbml.SBase sbase,
                                   java.util.Collection<java.lang.Object[]> resources)
```

:   **Parameters:**: `sbase` -: `resources` -


---


|  |  |  |  |  |  |  |  |  |  |
| --- | --- | --- | --- | --- | --- | --- | --- | --- | --- |
| |  |  |  |  |  |  |  | | --- | --- | --- | --- | --- | --- | --- | | **Overview** | **Package** | **Class** | **Tree** | **Deprecated** | **Index** | **Help** | | |  |
| PREV CLASS   **NEXT CLASS** | **FRAMES**    **NO FRAMES**     **All Classes** |
| SUMMARY: NESTED | FIELD | CONSTR | METHOD | DETAIL: FIELD | CONSTR | METHOD |


---
