# Supplementary material for: Path2Models: large-scale generation of computational models from biochemical pathway maps
Source: BMC Syst Biol. 2013 Nov 1;7:116. doi: 10.1186/1752-0509-7-116 (PMC4228421; doi:10.1186/1752-0509-7-116)
Supplement: Additional file 2 — Provided as an additional file and through labarchives, DOI:10.6070/H4WH2MX0. [file 1752-0509-7-116-S2.zip › Subliminal Toolbox v2/doc/mcisb-subliminal-lite/org/mcisb/subliminal_lite/kegg/KeggUtils.html]

KeggUtils


---


|  |  |  |  |  |  |  |  |  |  |
| --- | --- | --- | --- | --- | --- | --- | --- | --- | --- |
| |  |  |  |  |  |  |  | | --- | --- | --- | --- | --- | --- | --- | | **Overview** | **Package** | **Class** | **Tree** | **Deprecated** | **Index** | **Help** | | |  |
| **PREV CLASS**   **NEXT CLASS** | **FRAMES**    **NO FRAMES**     **All Classes** |
| SUMMARY: NESTED | FIELD | CONSTR | METHOD | DETAIL: FIELD | CONSTR | METHOD |


---


## org.mcisb.subliminal\_lite.kegg Class KeggUtils

```
java.lang.Object
  org.mcisb.subliminal_lite.kegg.KeggUtils
```

---

``` public class KeggUtils extends java.lang.Object ```

**Author:**
:   Neil Swainston

---

| **Constructor Summary** | |
| --- | --- |
| `KeggUtils()` |


| **Method Summary** | |
| --- | --- |
| `static java.lang.String[]` | `getOrganismIds()` |
| `static java.lang.String` | `getOrganismName(java.lang.String keggOrganismId)` |
| `static java.lang.String` | `getTaxonomyId(java.lang.String keggOrganismId)` |

| **Methods inherited from class java.lang.Object** |
| --- |
| `clone, equals, finalize, getClass, hashCode, notify, notifyAll, toString, wait, wait, wait` |

| **Constructor Detail** |
| --- |

### KeggUtils

```
public KeggUtils()
```


| **Method Detail** |
| --- |

### getOrganismIds

```
public static java.lang.String[] getOrganismIds()
                                         throws java.io.IOException
```

:   **Returns:**: String[] **Throws:**: `java.io.IOException`

---


### getTaxonomyId

```
public static java.lang.String getTaxonomyId(java.lang.String keggOrganismId)
                                      throws java.io.IOException
```

:   **Parameters:**: `keggOrganismId` - **Returns:**: String **Throws:**: `java.io.IOException`

---


### getOrganismName

```
public static java.lang.String getOrganismName(java.lang.String keggOrganismId)
                                        throws java.io.IOException
```

:   **Parameters:**: `keggOrganismId` - **Returns:**: String **Throws:**: `java.io.IOException`


---


|  |  |  |  |  |  |  |  |  |  |
| --- | --- | --- | --- | --- | --- | --- | --- | --- | --- |
| |  |  |  |  |  |  |  | | --- | --- | --- | --- | --- | --- | --- | | **Overview** | **Package** | **Class** | **Tree** | **Deprecated** | **Index** | **Help** | | |  |
| **PREV CLASS**   **NEXT CLASS** | **FRAMES**    **NO FRAMES**     **All Classes** |
| SUMMARY: NESTED | FIELD | CONSTR | METHOD | DETAIL: FIELD | CONSTR | METHOD |


---
