# Supplementary material for: Path2Models: large-scale generation of computational models from biochemical pathway maps
Source: BMC Syst Biol. 2013 Nov 1;7:116. doi: 10.1186/1752-0509-7-116 (PMC4228421; doi:10.1186/1752-0509-7-116)
Supplement: Additional file 2 — Provided as an additional file and through labarchives, DOI:10.6070/H4WH2MX0. [file 1752-0509-7-116-S2.zip › Subliminal Toolbox v2/doc/mcisb-subliminal-lite/org/mcisb/subliminal_lite/mnxref/MxnRefReactionUtils.html]

MxnRefReactionUtils


---


|  |  |  |  |  |  |  |  |  |  |
| --- | --- | --- | --- | --- | --- | --- | --- | --- | --- |
| |  |  |  |  |  |  |  | | --- | --- | --- | --- | --- | --- | --- | | **Overview** | **Package** | **Class** | **Tree** | **Deprecated** | **Index** | **Help** | | |  |
| **PREV CLASS**   **NEXT CLASS** | **FRAMES**    **NO FRAMES**     **All Classes** |
| SUMMARY: NESTED | FIELD | CONSTR | METHOD | DETAIL: FIELD | CONSTR | METHOD |


---


## org.mcisb.subliminal\_lite.mnxref Class MxnRefReactionUtils

```
java.lang.Object
  org.mcisb.subliminal_lite.mnxref.MxnRefUtils
      org.mcisb.subliminal_lite.mnxref.MxnRefReactionUtils
```

---

``` public class MxnRefReactionUtils extends MxnRefUtils ```

**Author:**
:   Neil Swainston

---

| **Nested Class Summary** | |
| --- | --- |

| **Nested classes/interfaces inherited from class org.mcisb.subliminal\_lite.mnxref.MxnRefUtils** |
| --- |
| `MxnRefUtils.Evidence` |


| **Field Summary** | |
| --- | --- |

| **Fields inherited from class org.mcisb.subliminal\_lite.mnxref.MxnRefUtils** |
| --- |
| `mxnRefIdToXrefIds` |


| **Method Summary** | |
| --- | --- |
| `boolean` | `getBalance(java.lang.String id)` |
| `java.lang.String` | `getDescription(java.lang.String id)` |
| `java.util.Collection<java.lang.String>` | `getEC(java.lang.String id)` |
| `java.lang.String` | `getEquation(java.lang.String id)` |
| `static MxnRefReactionUtils` | `getInstance()` |

| **Methods inherited from class org.mcisb.subliminal\_lite.mnxref.MxnRefUtils** |
| --- |
| `getData, getEvidence, getMxnRefId, getXrefIds, getXrefIds, initXrefs` |

| **Methods inherited from class java.lang.Object** |
| --- |
| `clone, equals, finalize, getClass, hashCode, notify, notifyAll, toString, wait, wait, wait` |

| **Method Detail** |
| --- |

### getInstance

```
public static MxnRefReactionUtils getInstance()
                                       throws java.net.MalformedURLException
```

:   **Returns:**: MxnRefChemUtils **Throws:**: `java.net.MalformedURLException`

---


### getEquation

```
public java.lang.String getEquation(java.lang.String id)
                             throws java.io.IOException,
                                    javax.xml.stream.XMLStreamException
```

:   **Parameters:**: `id` - **Returns:**: String **Throws:**: `java.io.IOException`: `javax.xml.stream.XMLStreamException`

---


### getDescription

```
public java.lang.String getDescription(java.lang.String id)
                                throws java.io.IOException,
                                       javax.xml.stream.XMLStreamException
```

:   **Parameters:**: `id` - **Returns:**: String **Throws:**: `java.io.IOException`: `javax.xml.stream.XMLStreamException`

---


### getBalance

```
public boolean getBalance(java.lang.String id)
                   throws java.io.IOException,
                          javax.xml.stream.XMLStreamException
```

:   **Parameters:**: `id` - **Returns:**: boolean **Throws:**: `java.io.IOException`: `javax.xml.stream.XMLStreamException`

---


### getEC

```
public java.util.Collection<java.lang.String> getEC(java.lang.String id)
                                             throws java.io.IOException,
                                                    javax.xml.stream.XMLStreamException
```

:   **Parameters:**: `id` - **Returns:**: Collection **Throws:**: `java.io.IOException`: `javax.xml.stream.XMLStreamException`


---


|  |  |  |  |  |  |  |  |  |  |
| --- | --- | --- | --- | --- | --- | --- | --- | --- | --- |
| |  |  |  |  |  |  |  | | --- | --- | --- | --- | --- | --- | --- | | **Overview** | **Package** | **Class** | **Tree** | **Deprecated** | **Index** | **Help** | | |  |
| **PREV CLASS**   **NEXT CLASS** | **FRAMES**    **NO FRAMES**     **All Classes** |
| SUMMARY: NESTED | FIELD | CONSTR | METHOD | DETAIL: FIELD | CONSTR | METHOD |


---
