# Supplementary material for: Path2Models: large-scale generation of computational models from biochemical pathway maps
Source: BMC Syst Biol. 2013 Nov 1;7:116. doi: 10.1186/1752-0509-7-116 (PMC4228421; doi:10.1186/1752-0509-7-116)
Supplement: Additional file 2 — Provided as an additional file and through labarchives, DOI:10.6070/H4WH2MX0. [file 1752-0509-7-116-S2.zip › Subliminal Toolbox v2/doc/mcisb-subliminal-lite/org/mcisb/subliminal_lite/mnxref/MxnRefReactionUtilsTest.html]

MxnRefReactionUtilsTest


---


|  |  |  |  |  |  |  |  |  |  |
| --- | --- | --- | --- | --- | --- | --- | --- | --- | --- |
| |  |  |  |  |  |  |  | | --- | --- | --- | --- | --- | --- | --- | | **Overview** | **Package** | **Class** | **Tree** | **Deprecated** | **Index** | **Help** | | |  |
| **PREV CLASS**   **NEXT CLASS** | **FRAMES**    **NO FRAMES**     **All Classes** |
| SUMMARY: NESTED | FIELD | CONSTR | METHOD | DETAIL: FIELD | CONSTR | METHOD |


---


## org.mcisb.subliminal\_lite.mnxref Class MxnRefReactionUtilsTest

```
java.lang.Object
  org.mcisb.subliminal_lite.mnxref.MxnRefReactionUtilsTest
```

---

``` public class MxnRefReactionUtilsTest extends java.lang.Object ```

**Author:**
:   Neil Swainston

---

| **Constructor Summary** | |
| --- | --- |
| `MxnRefReactionUtilsTest()` |


| **Method Summary** | |
| --- | --- |
| `void` | `getName()` |

| **Methods inherited from class java.lang.Object** |
| --- |
| `clone, equals, finalize, getClass, hashCode, notify, notifyAll, toString, wait, wait, wait` |

| **Constructor Detail** |
| --- |

### MxnRefReactionUtilsTest

```
public MxnRefReactionUtilsTest()
```


| **Method Detail** |
| --- |

### getName

```
public void getName()
             throws java.io.IOException,
                    javax.xml.stream.XMLStreamException
```

:   **Throws:**: `java.io.IOException`: `javax.xml.stream.XMLStreamException`


---


|  |  |  |  |  |  |  |  |  |  |
| --- | --- | --- | --- | --- | --- | --- | --- | --- | --- |
| |  |  |  |  |  |  |  | | --- | --- | --- | --- | --- | --- | --- | | **Overview** | **Package** | **Class** | **Tree** | **Deprecated** | **Index** | **Help** | | |  |
| **PREV CLASS**   **NEXT CLASS** | **FRAMES**    **NO FRAMES**     **All Classes** |
| SUMMARY: NESTED | FIELD | CONSTR | METHOD | DETAIL: FIELD | CONSTR | METHOD |


---
