# Supplementary material for: Path2Models: large-scale generation of computational models from biochemical pathway maps
Source: BMC Syst Biol. 2013 Nov 1;7:116. doi: 10.1186/1752-0509-7-116 (PMC4228421; doi:10.1186/1752-0509-7-116)
Supplement: Additional file 2 — Provided as an additional file and through labarchives, DOI:10.6070/H4WH2MX0. [file 1752-0509-7-116-S2.zip › Subliminal Toolbox v2/doc/mcisb-subliminal-lite/org/mcisb/subliminal_lite/mnxref/MxnRefUtils.Evidence.html]

MxnRefUtils.Evidence


---


|  |  |  |  |  |  |  |  |  |  |
| --- | --- | --- | --- | --- | --- | --- | --- | --- | --- |
| |  |  |  |  |  |  |  | | --- | --- | --- | --- | --- | --- | --- | | **Overview** | **Package** | **Class** | **Tree** | **Deprecated** | **Index** | **Help** | | |  |
| **PREV CLASS**   NEXT CLASS | **FRAMES**    **NO FRAMES**     **All Classes** |
| SUMMARY: NESTED | ENUM CONSTANTS | FIELD | METHOD | DETAIL: ENUM CONSTANTS | FIELD | METHOD |


---


## org.mcisb.subliminal\_lite.mnxref Enum MxnRefUtils.Evidence

```
java.lang.Object
  java.lang.Enum<MxnRefUtils.Evidence>
      org.mcisb.subliminal_lite.mnxref.MxnRefUtils.Evidence
```

**All Implemented Interfaces:**: java.io.Serializable, java.lang.Comparable<MxnRefUtils.Evidence>

**Enclosing class:**: MxnRefUtils

---

``` public static enum MxnRefUtils.Evidence extends java.lang.Enum<MxnRefUtils.Evidence> ```

**Author:**
:   Neil Swainston

---

| **Enum Constant Summary** | |
| --- | --- |
| `identity` |
| `inferred` |
| `structural` |


| **Method Summary** | |
| --- | --- |
| `static MxnRefUtils.Evidence` | `valueOf(java.lang.String name)`             Returns the enum constant of this type with the specified name. |
| `static MxnRefUtils.Evidence[]` | `values()`             Returns an array containing the constants of this enum type, in the order they are declared. |

| **Methods inherited from class java.lang.Enum** |
| --- |
| `clone, compareTo, equals, finalize, getDeclaringClass, hashCode, name, ordinal, toString, valueOf` |

| **Methods inherited from class java.lang.Object** |
| --- |
| `getClass, notify, notifyAll, wait, wait, wait` |

| **Enum Constant Detail** |
| --- |

### identity

```
public static final MxnRefUtils.Evidence identity
```

---


### inferred

```
public static final MxnRefUtils.Evidence inferred
```

---


### structural

```
public static final MxnRefUtils.Evidence structural
```


| **Method Detail** |
| --- |

### values

```
public static MxnRefUtils.Evidence[] values()
```

:   Returns an array containing the constants of this enum type, in
    the order they are declared. This method may be used to iterate
    over the constants as follows:

    ```
    for (MxnRefUtils.Evidence c : MxnRefUtils.Evidence.values())
        System.out.println(c);
    ```

    :   **Returns:**: an array containing the constants of this enum type, in the order they are declared

---


### valueOf

```
public static MxnRefUtils.Evidence valueOf(java.lang.String name)
```

:   Returns the enum constant of this type with the specified name.
    The string must match *exactly* an identifier used to declare an
    enum constant in this type. (Extraneous whitespace characters are
    not permitted.)

    :   **Parameters:**: `name` - the name of the enum constant to be returned. **Returns:**: the enum constant with the specified name **Throws:**: `java.lang.IllegalArgumentException` - if this enum type has no constant with the specified name: `java.lang.NullPointerException` - if the argument is null


---


|  |  |  |  |  |  |  |  |  |  |
| --- | --- | --- | --- | --- | --- | --- | --- | --- | --- |
| |  |  |  |  |  |  |  | | --- | --- | --- | --- | --- | --- | --- | | **Overview** | **Package** | **Class** | **Tree** | **Deprecated** | **Index** | **Help** | | |  |
| **PREV CLASS**   NEXT CLASS | **FRAMES**    **NO FRAMES**     **All Classes** |
| SUMMARY: NESTED | ENUM CONSTANTS | FIELD | METHOD | DETAIL: ENUM CONSTANTS | FIELD | METHOD |


---
