# Supplementary material for: Path2Models: large-scale generation of computational models from biochemical pathway maps
Source: BMC Syst Biol. 2013 Nov 1;7:116. doi: 10.1186/1752-0509-7-116 (PMC4228421; doi:10.1186/1752-0509-7-116)
Supplement: Additional file 2 — Provided as an additional file and through labarchives, DOI:10.6070/H4WH2MX0. [file 1752-0509-7-116-S2.zip › Subliminal Toolbox v2/doc/mcisb-subliminal-lite/org/mcisb/subliminal_lite/mnxref/MxnRefUtils.html]

MxnRefUtils


---


|  |  |  |  |  |  |  |  |  |  |
| --- | --- | --- | --- | --- | --- | --- | --- | --- | --- |
| |  |  |  |  |  |  |  | | --- | --- | --- | --- | --- | --- | --- | | **Overview** | **Package** | **Class** | **Tree** | **Deprecated** | **Index** | **Help** | | |  |
| **PREV CLASS**   **NEXT CLASS** | **FRAMES**    **NO FRAMES**     **All Classes** |
| SUMMARY: NESTED | FIELD | CONSTR | METHOD | DETAIL: FIELD | CONSTR | METHOD |


---


## org.mcisb.subliminal\_lite.mnxref Class MxnRefUtils

```
java.lang.Object
  org.mcisb.subliminal_lite.mnxref.MxnRefUtils
```

**Direct Known Subclasses:**: MxnRefChemUtils, MxnRefReactionUtils

---

``` public abstract class MxnRefUtils extends java.lang.Object ```

**Author:**
:   Neil Swainston

---

| **Nested Class Summary** | |
| --- | --- |
| `static class` | `MxnRefUtils.Evidence` |


| **Field Summary** | |
| --- | --- |
| `protected  java.util.Map<java.lang.String,java.util.Map<java.lang.String,java.util.Collection<java.lang.String>>>` | `mxnRefIdToXrefIds` |


| **Constructor Summary** | |
| --- | --- |
| `protected` | `MxnRefUtils(java.lang.String mxnRefIdRegExp, java.net.URL xrefUrl)` |


| **Method Summary** | |
| --- | --- |
| `protected  java.lang.String` | `getData(java.lang.String id, int column, java.net.URL dataUrl)` |
| `MxnRefUtils.Evidence` | `getEvidence(java.lang.String xrefId)` |
| `java.lang.String` | `getMxnRefId(java.lang.String id)` |
| `java.util.Map<java.lang.String,java.util.Collection<java.lang.String>>` | `getXrefIds(java.lang.String id)` |
| `java.util.Collection<java.lang.String>` | `getXrefIds(java.lang.String id, java.lang.String source)` |
| `protected  void` | `initXrefs()` |

| **Methods inherited from class java.lang.Object** |
| --- |
| `clone, equals, finalize, getClass, hashCode, notify, notifyAll, toString, wait, wait, wait` |

| **Field Detail** |
| --- |

### mxnRefIdToXrefIds

```
protected java.util.Map<java.lang.String,java.util.Map<java.lang.String,java.util.Collection<java.lang.String>>> mxnRefIdToXrefIds
```


| **Constructor Detail** |
| --- |

### MxnRefUtils

```
protected MxnRefUtils(java.lang.String mxnRefIdRegExp,
                      java.net.URL xrefUrl)
```

**Parameters:**: `mxnRefIdRegExp` -: `xrefUrl` -


| **Method Detail** |
| --- |

### getMxnRefId

```
public java.lang.String getMxnRefId(java.lang.String id)
                             throws java.io.IOException,
                                    javax.xml.stream.XMLStreamException
```

:   **Parameters:**: `id` - **Returns:**: String **Throws:**: `java.io.IOException`: `javax.xml.stream.XMLStreamException`

---


### getXrefIds

```
public java.util.Map<java.lang.String,java.util.Collection<java.lang.String>> getXrefIds(java.lang.String id)
                                                                                  throws java.io.IOException,
                                                                                         javax.xml.stream.XMLStreamException
```

:   **Parameters:**: `id` - **Returns:**: Map> **Throws:**: `java.io.IOException`: `javax.xml.stream.XMLStreamException`

---


### getXrefIds

```
public java.util.Collection<java.lang.String> getXrefIds(java.lang.String id,
                                                         java.lang.String source)
                                                  throws java.io.IOException,
                                                         javax.xml.stream.XMLStreamException
```

:   **Parameters:**: `id` - **Returns:**: Collection **Throws:**: `java.io.IOException`: `javax.xml.stream.XMLStreamException`

---


### getEvidence

```
public MxnRefUtils.Evidence getEvidence(java.lang.String xrefId)
                                 throws java.io.IOException,
                                        javax.xml.stream.XMLStreamException
```

:   **Parameters:**: `xrefId` - **Returns:**: Evidence **Throws:**: `java.io.IOException`: `javax.xml.stream.XMLStreamException`

---


### getData

```
protected java.lang.String getData(java.lang.String id,
                                   int column,
                                   java.net.URL dataUrl)
                            throws java.io.IOException,
                                   javax.xml.stream.XMLStreamException
```

:   **Parameters:**: `id` -: `column` -: `dataUrl` - **Returns:**: String **Throws:**: `java.io.IOException`: `javax.xml.stream.XMLStreamException`

---


### initXrefs

```
protected void initXrefs()
                  throws java.io.IOException,
                         javax.xml.stream.XMLStreamException
```

:   **Throws:**: `java.io.IOException`: `javax.xml.stream.XMLStreamException`


---


|  |  |  |  |  |  |  |  |  |  |
| --- | --- | --- | --- | --- | --- | --- | --- | --- | --- |
| |  |  |  |  |  |  |  | | --- | --- | --- | --- | --- | --- | --- | | **Overview** | **Package** | **Class** | **Tree** | **Deprecated** | **Index** | **Help** | | |  |
| **PREV CLASS**   **NEXT CLASS** | **FRAMES**    **NO FRAMES**     **All Classes** |
| SUMMARY: NESTED | FIELD | CONSTR | METHOD | DETAIL: FIELD | CONSTR | METHOD |


---
