# Supplementary material for: Path2Models: large-scale generation of computational models from biochemical pathway maps
Source: BMC Syst Biol. 2013 Nov 1;7:116. doi: 10.1186/1752-0509-7-116 (PMC4228421; doi:10.1186/1752-0509-7-116)
Supplement: Additional file 2 — Provided as an additional file and through labarchives, DOI:10.6070/H4WH2MX0. [file 1752-0509-7-116-S2.zip › Subliminal Toolbox v2/doc/mcisb-subliminal-lite/org/mcisb/subliminal_lite/model/BiomassExtracter.html]

BiomassExtracter


---


|  |  |  |  |  |  |  |  |  |  |
| --- | --- | --- | --- | --- | --- | --- | --- | --- | --- |
| |  |  |  |  |  |  |  | | --- | --- | --- | --- | --- | --- | --- | | **Overview** | **Package** | **Class** | **Tree** | **Deprecated** | **Index** | **Help** | | |  |
| PREV CLASS   **NEXT CLASS** | **FRAMES**    **NO FRAMES**     **All Classes** |
| SUMMARY: NESTED | FIELD | CONSTR | METHOD | DETAIL: FIELD | CONSTR | METHOD |


---


## org.mcisb.subliminal\_lite.model Class BiomassExtracter

```
java.lang.Object
  org.mcisb.subliminal_lite.Extracter
      org.mcisb.subliminal_lite.model.BiomassExtracter
```

---

``` public class BiomassExtracter extends Extracter ```

**Author:**
:   Neil Swainston

---

| **Field Summary** | |
| --- | --- |

| **Fields inherited from class org.mcisb.subliminal\_lite.Extracter** |
| --- |
| `BIOMASS_COMPARTMENT_ID, DEFAULT_COMPARTMENT_ID, EXTRACELLULAR_COMPARTMENT_ID` |


| **Constructor Summary** | |
| --- | --- |
| `BiomassExtracter()` |


| **Method Summary** | |
| --- | --- |
| `static void` | `run(org.sbml.jsbml.SBMLDocument document)` |

| **Methods inherited from class org.mcisb.subliminal\_lite.Extracter** |
| --- |
| `addCompartment, addDefaultCompartment, addEnzyme, addEnzymes, addReaction, addResources, addSpecies, addSpecies, initDocument` |

| **Methods inherited from class java.lang.Object** |
| --- |
| `clone, equals, finalize, getClass, hashCode, notify, notifyAll, toString, wait, wait, wait` |

| **Constructor Detail** |
| --- |

### BiomassExtracter

```
public BiomassExtracter()
```


| **Method Detail** |
| --- |

### run

```
public static void run(org.sbml.jsbml.SBMLDocument document)
                throws java.lang.Exception
```

:   **Parameters:**: `document` - **Throws:**: `java.lang.Exception`


---


|  |  |  |  |  |  |  |  |  |  |
| --- | --- | --- | --- | --- | --- | --- | --- | --- | --- |
| |  |  |  |  |  |  |  | | --- | --- | --- | --- | --- | --- | --- | | **Overview** | **Package** | **Class** | **Tree** | **Deprecated** | **Index** | **Help** | | |  |
| PREV CLASS   **NEXT CLASS** | **FRAMES**    **NO FRAMES**     **All Classes** |
| SUMMARY: NESTED | FIELD | CONSTR | METHOD | DETAIL: FIELD | CONSTR | METHOD |


---
