# Supplementary material for: Path2Models: large-scale generation of computational models from biochemical pathway maps
Source: BMC Syst Biol. 2013 Nov 1;7:116. doi: 10.1186/1752-0509-7-116 (PMC4228421; doi:10.1186/1752-0509-7-116)
Supplement: Additional file 2 — Provided as an additional file and through labarchives, DOI:10.6070/H4WH2MX0. [file 1752-0509-7-116-S2.zip › Subliminal Toolbox v2/doc/mcisb-subliminal-lite/org/mcisb/subliminal_lite/model/package-tree.html]

org.mcisb.subliminal\_lite.model Class Hierarchy


---


|  |  |  |  |  |  |  |  |  |  |
| --- | --- | --- | --- | --- | --- | --- | --- | --- | --- |
| |  |  |  |  |  |  |  | | --- | --- | --- | --- | --- | --- | --- | | **Overview** | **Package** | Class | **Tree** | **Deprecated** | **Index** | **Help** | | |  |
| **PREV**   **NEXT** | **FRAMES**    **NO FRAMES**     **All Classes** |


---


## Hierarchy For Package org.mcisb.subliminal\_lite.model

**Package Hierarchies:**: All Packages

---

## Class Hierarchy

- java.lang.Object
  - org.mcisb.subliminal\_lite.**Extracter**
    - org.mcisb.subliminal\_lite.model.**BiomassExtracter**- org.mcisb.subliminal\_lite.model.**CobraFormatter**- org.mcisb.subliminal\_lite.model.**TransportExtracter**- org.mcisb.subliminal\_lite.model.**FluxBoundsGenerater**- org.mcisb.subliminal\_lite.model.**GeneAssociationGenerater**- org.mcisb.subliminal\_lite.model.**ModelGenerater**- org.mcisb.subliminal\_lite.model.**XmlFormatter**

---


|  |  |  |  |  |  |  |  |  |  |
| --- | --- | --- | --- | --- | --- | --- | --- | --- | --- |
| |  |  |  |  |  |  |  | | --- | --- | --- | --- | --- | --- | --- | | **Overview** | **Package** | Class | **Tree** | **Deprecated** | **Index** | **Help** | | |  |
| **PREV**   **NEXT** | **FRAMES**    **NO FRAMES**     **All Classes** |


---
