# Supplementary material for: Path2Models: large-scale generation of computational models from biochemical pathway maps
Source: BMC Syst Biol. 2013 Nov 1;7:116. doi: 10.1186/1752-0509-7-116 (PMC4228421; doi:10.1186/1752-0509-7-116)
Supplement: Additional file 2 — Provided as an additional file and through labarchives, DOI:10.6070/H4WH2MX0. [file 1752-0509-7-116-S2.zip › Subliminal Toolbox v2/doc/mcisb-subliminal-lite/org/mcisb/subliminal_lite/model/XmlFormatter.html]

XmlFormatter


---


|  |  |  |  |  |  |  |  |  |  |
| --- | --- | --- | --- | --- | --- | --- | --- | --- | --- |
| |  |  |  |  |  |  |  | | --- | --- | --- | --- | --- | --- | --- | | **Overview** | **Package** | **Class** | **Tree** | **Deprecated** | **Index** | **Help** | | |  |
| **PREV CLASS**   NEXT CLASS | **FRAMES**    **NO FRAMES**     **All Classes** |
| SUMMARY: NESTED | FIELD | CONSTR | METHOD | DETAIL: FIELD | CONSTR | METHOD |


---


## org.mcisb.subliminal\_lite.model Class XmlFormatter

```
java.lang.Object
  org.mcisb.subliminal_lite.model.XmlFormatter
```

---

``` public class XmlFormatter extends java.lang.Object ```

**Author:**
:   neilswainston

---

| **Method Summary** | |
| --- | --- |
| `static XmlFormatter` | `getInstance()` |
| `static void` | `main(java.lang.String[] args)` |
| `void` | `write(java.io.File inFile, java.io.File outFile)` |
| `void` | `write(org.sbml.jsbml.SBMLDocument document, java.io.File outFile)` |

| **Methods inherited from class java.lang.Object** |
| --- |
| `clone, equals, finalize, getClass, hashCode, notify, notifyAll, toString, wait, wait, wait` |

| **Method Detail** |
| --- |

### getInstance

```
public static XmlFormatter getInstance()
                                throws javax.xml.transform.TransformerConfigurationException,
                                       javax.xml.transform.TransformerFactoryConfigurationError,
                                       java.io.IOException
```

:   **Returns:**: XmlFormatter **Throws:**: `javax.xml.transform.TransformerFactoryConfigurationError`: `javax.xml.transform.TransformerConfigurationException`: `java.io.IOException`

---


### write

```
public void write(org.sbml.jsbml.SBMLDocument document,
                  java.io.File outFile)
           throws javax.xml.transform.TransformerFactoryConfigurationError,
                  javax.xml.transform.TransformerException,
                  org.sbml.jsbml.SBMLException,
                  javax.xml.stream.XMLStreamException,
                  java.io.IOException
```

:   **Parameters:**: `document` -: `outFile` - **Throws:**: `javax.xml.transform.TransformerFactoryConfigurationError`: `javax.xml.transform.TransformerException`: `org.sbml.jsbml.SBMLException`: `javax.xml.stream.XMLStreamException`: `KeggUtils.getOrganismName(` - keggOrganismId ): `java.io.IOException`

---


### write

```
public void write(java.io.File inFile,
                  java.io.File outFile)
           throws javax.xml.transform.TransformerFactoryConfigurationError,
                  javax.xml.transform.TransformerException,
                  org.sbml.jsbml.SBMLException,
                  javax.xml.stream.XMLStreamException,
                  java.io.IOException
```

:   **Parameters:**: `inFile` -: `outFile` - **Throws:**: `javax.xml.transform.TransformerFactoryConfigurationError`: `javax.xml.transform.TransformerException`: `org.sbml.jsbml.SBMLException`: `javax.xml.stream.XMLStreamException`: `java.io.IOException`

---


### main

```
public static void main(java.lang.String[] args)
                 throws org.sbml.jsbml.SBMLException,
                        javax.xml.transform.TransformerConfigurationException,
                        javax.xml.transform.TransformerFactoryConfigurationError,
                        javax.xml.transform.TransformerException,
                        javax.xml.stream.XMLStreamException,
                        java.io.IOException
```

:   **Parameters:**: `args` - **Throws:**: `java.io.IOException`: `javax.xml.stream.XMLStreamException`: `javax.xml.transform.TransformerException`: `javax.xml.transform.TransformerFactoryConfigurationError`: `javax.xml.transform.TransformerConfigurationException`: `org.sbml.jsbml.SBMLException`


---


|  |  |  |  |  |  |  |  |  |  |
| --- | --- | --- | --- | --- | --- | --- | --- | --- | --- |
| |  |  |  |  |  |  |  | | --- | --- | --- | --- | --- | --- | --- | | **Overview** | **Package** | **Class** | **Tree** | **Deprecated** | **Index** | **Help** | | |  |
| **PREV CLASS**   NEXT CLASS | **FRAMES**    **NO FRAMES**     **All Classes** |
| SUMMARY: NESTED | FIELD | CONSTR | METHOD | DETAIL: FIELD | CONSTR | METHOD |


---
