# Supplementary material for: Path2Models: large-scale generation of computational models from biochemical pathway maps
Source: BMC Syst Biol. 2013 Nov 1;7:116. doi: 10.1186/1752-0509-7-116 (PMC4228421; doi:10.1186/1752-0509-7-116)
Supplement: Additional file 2 — Provided as an additional file and through labarchives, DOI:10.6070/H4WH2MX0. [file 1752-0509-7-116-S2.zip › Subliminal Toolbox v2/doc/mcisb-subliminal-lite/org/mcisb/subliminal_lite/package-frame.html]

org.mcisb.subliminal\_lite


org.mcisb.subliminal\_lite

|  |
| --- |
| Classes    Extracter   Path2ModelsReconstructionGenerator   SubliminalUtils   SubliminalUtilsTest |
