# Supplementary material for: Path2Models: large-scale generation of computational models from biochemical pathway maps
Source: BMC Syst Biol. 2013 Nov 1;7:116. doi: 10.1186/1752-0509-7-116 (PMC4228421; doi:10.1186/1752-0509-7-116)
Supplement: Additional file 2 — Provided as an additional file and through labarchives, DOI:10.6070/H4WH2MX0. [file 1752-0509-7-116-S2.zip › Subliminal Toolbox v2/doc/mcisb-subliminal-lite/org/mcisb/subliminal_lite/sbml/SbmlFactory.html]

SbmlFactory


---


|  |  |  |  |  |  |  |  |  |  |
| --- | --- | --- | --- | --- | --- | --- | --- | --- | --- |
| |  |  |  |  |  |  |  | | --- | --- | --- | --- | --- | --- | --- | | **Overview** | **Package** | **Class** | **Tree** | **Deprecated** | **Index** | **Help** | | |  |
| PREV CLASS   NEXT CLASS | **FRAMES**    **NO FRAMES**     **All Classes** |
| SUMMARY: NESTED | FIELD | CONSTR | METHOD | DETAIL: FIELD | CONSTR | METHOD |


---


## org.mcisb.subliminal\_lite.sbml Class SbmlFactory

```
java.lang.Object
  org.mcisb.subliminal_lite.sbml.SbmlFactory
```

---

``` public class SbmlFactory extends java.lang.Object ```

**Author:**
:   Neil Swainston

---

| **Method Summary** | |
| --- | --- |
| `static SbmlFactory` | `getInstance()` |
| `org.sbml.jsbml.Reaction` | `getReaction(java.lang.String reactionId, java.lang.String compartmentId)` |
| `org.sbml.jsbml.Species` | `getSpecies(java.lang.String id, java.lang.String compartment)` |
| `void` | `unregister()` |

| **Methods inherited from class java.lang.Object** |
| --- |
| `clone, equals, finalize, getClass, hashCode, notify, notifyAll, toString, wait, wait, wait` |

| **Method Detail** |
| --- |

### getInstance

```
public static SbmlFactory getInstance()
                               throws java.net.MalformedURLException
```

:   **Returns:**: SbmlFactory **Throws:**: `java.net.MalformedURLException`

---


### unregister

```
public void unregister()
```

---


### getSpecies

```
public org.sbml.jsbml.Species getSpecies(java.lang.String id,
                                         java.lang.String compartment)
                                  throws java.io.IOException,
                                         javax.xml.stream.XMLStreamException
```

:   **Parameters:**: `id` -: `compartment` - **Returns:**: Species **Throws:**: `java.io.IOException`: `javax.xml.stream.XMLStreamException`

---


### getReaction

```
public org.sbml.jsbml.Reaction getReaction(java.lang.String reactionId,
                                           java.lang.String compartmentId)
                                    throws java.io.IOException,
                                           javax.xml.stream.XMLStreamException
```

:   **Parameters:**: `reactionId` -: `compartmentId` - **Returns:**: Reaction **Throws:**: `java.io.IOException`: `javax.xml.stream.XMLStreamException`


---


|  |  |  |  |  |  |  |  |  |  |
| --- | --- | --- | --- | --- | --- | --- | --- | --- | --- |
| |  |  |  |  |  |  |  | | --- | --- | --- | --- | --- | --- | --- | | **Overview** | **Package** | **Class** | **Tree** | **Deprecated** | **Index** | **Help** | | |  |
| PREV CLASS   NEXT CLASS | **FRAMES**    **NO FRAMES**     **All Classes** |
| SUMMARY: NESTED | FIELD | CONSTR | METHOD | DETAIL: FIELD | CONSTR | METHOD |


---
