# Supplementary material for: Path2Models: large-scale generation of computational models from biochemical pathway maps
Source: BMC Syst Biol. 2013 Nov 1;7:116. doi: 10.1186/1752-0509-7-116 (PMC4228421; doi:10.1186/1752-0509-7-116)
Supplement: Additional file 2 — Provided as an additional file and through labarchives, DOI:10.6070/H4WH2MX0. [file 1752-0509-7-116-S2.zip › Subliminal Toolbox v2/doc/mcisb-subliminal-lite/org/mcisb/subliminal_lite/SubliminalUtils.html]

SubliminalUtils


---


|  |  |  |  |  |  |  |  |  |  |
| --- | --- | --- | --- | --- | --- | --- | --- | --- | --- |
| |  |  |  |  |  |  |  | | --- | --- | --- | --- | --- | --- | --- | | **Overview** | **Package** | **Class** | **Tree** | **Deprecated** | **Index** | **Help** | | |  |
| **PREV CLASS**   **NEXT CLASS** | **FRAMES**    **NO FRAMES**     **All Classes** |
| SUMMARY: NESTED | FIELD | CONSTR | METHOD | DETAIL: FIELD | CONSTR | METHOD |


---


## org.mcisb.subliminal\_lite Class SubliminalUtils

```
java.lang.Object
  org.mcisb.subliminal_lite.SubliminalUtils
```

---

``` public class SubliminalUtils extends java.lang.Object ```

**Author:**
:   Neil Swainston

---

| **Field Summary** | |
| --- | --- |
| `static java.lang.String` | `BIOMASS_REACTION` |
| `static java.lang.String` | `CHARGE` |
| `static java.lang.String` | `COLON` |
| `static double` | `DEFAULT_INITIAL_CONCENTRATION` |
| `static int` | `DEFAULT_LEVEL` |
| `static int` | `DEFAULT_VERSION` |
| `static java.lang.String` | `EMPTY_STRING` |
| `static int` | `FIRST` |
| `static java.lang.String` | `FORMULA` |
| `static java.lang.String` | `HYPHEN` |
| `static java.lang.String` | `INCHI` |
| `static java.lang.String` | `NON_WORD` |
| `static int` | `SBO_BIOCHEMICAL_REACTION` |
| `static int` | `SBO_COMPARTMENT` |
| `static int` | `SBO_OMITTED_PROCESS` |
| `static int` | `SBO_POLYPEPTIDE_CHAIN` |
| `static int` | `SBO_PROTEIN_COMPLEX` |
| `static int` | `SBO_SIMPLE_CHEMICAL` |
| `static int` | `SBO_TRANSPORT_REACTION` |
| `static java.lang.String` | `SMILES` |
| `static int` | `UNDEFINED_NUMBER` |
| `static java.lang.String` | `UNDERSCORE` |
| `static java.lang.String` | `WHITESPACE` |


| **Constructor Summary** | |
| --- | --- |
| `SubliminalUtils()` |


| **Method Summary** | |
| --- | --- |
| `protected static void` | `addCVTerm(org.sbml.jsbml.SBase sbase, java.lang.String resource, org.sbml.jsbml.CVTerm.Type type, org.sbml.jsbml.CVTerm.Qualifier qualifier)` |
| `static void` | `addCVTerms(org.sbml.jsbml.SBase sbase, java.util.Collection<java.lang.String> resources, org.sbml.jsbml.CVTerm.Type type, org.sbml.jsbml.CVTerm.Qualifier qualifier)` |
| `static void` | `addHistory(org.sbml.jsbml.SBMLDocument document)` |
| `static void` | `addNote(org.sbml.jsbml.SBase sbase, java.lang.String key, java.lang.Object value)` |
| `static void` | `delete(java.io.File file)` |
| `static java.lang.String` | `encodeUniProtSearchTerm(java.lang.String term)` |
| `static java.io.File` | `find(java.io.File root, java.lang.String name)` |
| `static java.lang.String` | `getCompartmentalisedId(java.lang.String id, java.lang.String compartmentId)` |
| `static java.lang.String` | `getDecompartmentalisedId(java.lang.String id, java.lang.String compartmentId)` |
| `static java.lang.String` | `getNormalisedId(java.lang.String id)` |
| `static java.util.Map<java.lang.String,java.lang.Object>` | `getNotes(org.sbml.jsbml.SBase sbase)` |
| `static java.util.List<java.lang.String>` | `getNoteValues(org.sbml.jsbml.SBase sbase, java.lang.String key)` |
| `static java.lang.String` | `getTaxonomyName(java.lang.String taxonomyId)` |
| `static java.lang.String` | `getUniqueId()` |
| `static double` | `parseStoichiometry(java.lang.String s)` |
| `static java.util.List<java.lang.String[]>` | `searchUniProt(java.lang.String query)` |
| `static void` | `setNotes(org.sbml.jsbml.SBase sbase, java.util.Map<java.lang.String,java.lang.Object> notes)` |
| `static java.lang.String` | `stripTags(java.lang.String tagged)` |
| `static java.lang.String` | `toString(java.util.Collection<?> values)` |
| `static void` | `untar(java.net.URL url, java.io.File destinationDirectory)` |

| **Methods inherited from class java.lang.Object** |
| --- |
| `clone, equals, finalize, getClass, hashCode, notify, notifyAll, toString, wait, wait, wait` |

| **Field Detail** |
| --- |

### FIRST

```
public static final int FIRST
```

**See Also:**: Constant Field Values

---


### DEFAULT\_LEVEL

```
public static final int DEFAULT_LEVEL
```

**See Also:**: Constant Field Values

---


### DEFAULT\_VERSION

```
public static final int DEFAULT_VERSION
```

**See Also:**: Constant Field Values

---


### DEFAULT\_INITIAL\_CONCENTRATION

```
public static final double DEFAULT_INITIAL_CONCENTRATION
```

**See Also:**: Constant Field Values

---


### SBO\_BIOCHEMICAL\_REACTION

```
public static final int SBO_BIOCHEMICAL_REACTION
```

**See Also:**: Constant Field Values

---


### SBO\_TRANSPORT\_REACTION

```
public static final int SBO_TRANSPORT_REACTION
```

**See Also:**: Constant Field Values

---


### SBO\_SIMPLE\_CHEMICAL

```
public static final int SBO_SIMPLE_CHEMICAL
```

**See Also:**: Constant Field Values

---


### SBO\_POLYPEPTIDE\_CHAIN

```
public static final int SBO_POLYPEPTIDE_CHAIN
```

**See Also:**: Constant Field Values

---


### SBO\_COMPARTMENT

```
public static final int SBO_COMPARTMENT
```

**See Also:**: Constant Field Values

---


### SBO\_PROTEIN\_COMPLEX

```
public static final int SBO_PROTEIN_COMPLEX
```

**See Also:**: Constant Field Values

---


### SBO\_OMITTED\_PROCESS

```
public static final int SBO_OMITTED_PROCESS
```

**See Also:**: Constant Field Values

---


### UNDEFINED\_NUMBER

```
public static final int UNDEFINED_NUMBER
```

**See Also:**: Constant Field Values

---


### BIOMASS\_REACTION

```
public static final java.lang.String BIOMASS_REACTION
```

**See Also:**: Constant Field Values

---


### FORMULA

```
public static final java.lang.String FORMULA
```

**See Also:**: Constant Field Values

---


### CHARGE

```
public static final java.lang.String CHARGE
```

**See Also:**: Constant Field Values

---


### INCHI

```
public static final java.lang.String INCHI
```

**See Also:**: Constant Field Values

---


### SMILES

```
public static final java.lang.String SMILES
```

**See Also:**: Constant Field Values

---


### HYPHEN

```
public static final java.lang.String HYPHEN
```

**See Also:**: Constant Field Values

---


### UNDERSCORE

```
public static final java.lang.String UNDERSCORE
```

**See Also:**: Constant Field Values

---


### EMPTY\_STRING

```
public static final java.lang.String EMPTY_STRING
```

**See Also:**: Constant Field Values

---


### COLON

```
public static final java.lang.String COLON
```

**See Also:**: Constant Field Values

---


### NON\_WORD

```
public static final java.lang.String NON_WORD
```

**See Also:**: Constant Field Values

---


### WHITESPACE

```
public static final java.lang.String WHITESPACE
```

**See Also:**: Constant Field Values


| **Constructor Detail** |
| --- |

### SubliminalUtils

```
public SubliminalUtils()
```


| **Method Detail** |
| --- |

### getTaxonomyName

```
public static java.lang.String getTaxonomyName(java.lang.String taxonomyId)
                                        throws java.io.IOException
```

:   **Parameters:**: `taxonomyId` - **Returns:**: String **Throws:**: `java.io.IOException`

---


### addHistory

```
public static void addHistory(org.sbml.jsbml.SBMLDocument document)
```

:   **Parameters:**: `document` -

---


### addCVTerm

```
protected static void addCVTerm(org.sbml.jsbml.SBase sbase,
                                java.lang.String resource,
                                org.sbml.jsbml.CVTerm.Type type,
                                org.sbml.jsbml.CVTerm.Qualifier qualifier)
```

:   **Parameters:**: `sbase` -: `resource` -: `type` -: `qualifier` -

---


### addCVTerms

```
public static void addCVTerms(org.sbml.jsbml.SBase sbase,
                              java.util.Collection<java.lang.String> resources,
                              org.sbml.jsbml.CVTerm.Type type,
                              org.sbml.jsbml.CVTerm.Qualifier qualifier)
```

:   **Parameters:**: `sbase` -: `resources` -: `type` -: `qualifier` -

---


### getUniqueId

```
public static java.lang.String getUniqueId()
```

:   **Returns:**: String

---


### getNormalisedId

```
public static java.lang.String getNormalisedId(java.lang.String id)
```

:   **Parameters:**: `id` - **Returns:**: String

---


### getCompartmentalisedId

```
public static java.lang.String getCompartmentalisedId(java.lang.String id,
                                                      java.lang.String compartmentId)
```

:   **Parameters:**: `id` -: `compartmentId` - **Returns:**: String

---


### getDecompartmentalisedId

```
public static java.lang.String getDecompartmentalisedId(java.lang.String id,
                                                        java.lang.String compartmentId)
```

:   **Parameters:**: `id` -: `compartmentId` - **Returns:**: String

---


### find

```
public static java.io.File find(java.io.File root,
                                java.lang.String name)
```

:   **Parameters:**: `root` -: `name` - **Returns:**: File

---


### stripTags

```
public static java.lang.String stripTags(java.lang.String tagged)
```

:   **Parameters:**: `tagged` - **Returns:**: String

---


### untar

```
public static void untar(java.net.URL url,
                         java.io.File destinationDirectory)
                  throws java.io.IOException
```

:   **Parameters:**: `url` -: `destinationDirectory` - **Throws:**: `java.io.IOException`

---


### searchUniProt

```
public static java.util.List<java.lang.String[]> searchUniProt(java.lang.String query)
                                                        throws java.lang.Exception
```

:   **Parameters:**: `query` - **Returns:**: List **Throws:**: `java.lang.Exception`

---


### encodeUniProtSearchTerm

```
public static java.lang.String encodeUniProtSearchTerm(java.lang.String term)
```

---


### setNotes

```
public static void setNotes(org.sbml.jsbml.SBase sbase,
                            java.util.Map<java.lang.String,java.lang.Object> notes)
```

:   **Parameters:**: `sbase` -: `notes` -

---


### addNote

```
public static void addNote(org.sbml.jsbml.SBase sbase,
                           java.lang.String key,
                           java.lang.Object value)
                    throws java.io.UnsupportedEncodingException,
                           javax.xml.stream.XMLStreamException
```

:   **Parameters:**: `sbase` -: `key` -: `value` - **Throws:**: `javax.xml.stream.XMLStreamException`: `java.io.UnsupportedEncodingException`

---


### getNotes

```
public static java.util.Map<java.lang.String,java.lang.Object> getNotes(org.sbml.jsbml.SBase sbase)
                                                                 throws java.io.UnsupportedEncodingException,
                                                                        javax.xml.stream.XMLStreamException
```

:   **Parameters:**: `sbase` - **Returns:**: Map **Throws:**: `javax.xml.stream.XMLStreamException`: `java.io.UnsupportedEncodingException`

---


### getNoteValues

```
public static java.util.List<java.lang.String> getNoteValues(org.sbml.jsbml.SBase sbase,
                                                             java.lang.String key)
                                                      throws java.io.UnsupportedEncodingException,
                                                             javax.xml.stream.XMLStreamException
```

:   **Parameters:**: `sbase` -: `key` - **Returns:**: Collection **Throws:**: `java.io.UnsupportedEncodingException`: `javax.xml.stream.XMLStreamException`

---


### parseStoichiometry

```
public static double parseStoichiometry(java.lang.String s)
```

:   **Parameters:**: `s` - **Returns:**: double

---


### toString

```
public static java.lang.String toString(java.util.Collection<?> values)
```

:   **Parameters:**: `values` - **Returns:**: String

---


### delete

```
public static void delete(java.io.File file)
```

:   **Parameters:**: `file` -


---


|  |  |  |  |  |  |  |  |  |  |
| --- | --- | --- | --- | --- | --- | --- | --- | --- | --- |
| |  |  |  |  |  |  |  | | --- | --- | --- | --- | --- | --- | --- | | **Overview** | **Package** | **Class** | **Tree** | **Deprecated** | **Index** | **Help** | | |  |
| **PREV CLASS**   **NEXT CLASS** | **FRAMES**    **NO FRAMES**     **All Classes** |
| SUMMARY: NESTED | FIELD | CONSTR | METHOD | DETAIL: FIELD | CONSTR | METHOD |


---
