# Supplementary material for: Path2Models: large-scale generation of computational models from biochemical pathway maps
Source: BMC Syst Biol. 2013 Nov 1;7:116. doi: 10.1186/1752-0509-7-116 (PMC4228421; doi:10.1186/1752-0509-7-116)
Supplement: Additional file 2 — Provided as an additional file and through labarchives, DOI:10.6070/H4WH2MX0. [file 1752-0509-7-116-S2.zip › Subliminal Toolbox v2/doc/mcisb-subliminal-lite/overview-frame.html]

Overview List


|  |
| --- |

|  |
| --- |
| All Classes Packages   org.mcisb.subliminal\_lite   org.mcisb.subliminal\_lite.kegg   org.mcisb.subliminal\_lite.merge   org.mcisb.subliminal\_lite.metacyc   org.mcisb.subliminal\_lite.mnxref   org.mcisb.subliminal\_lite.model   org.mcisb.subliminal\_lite.sbml   org.mcisb.subliminal\_lite.xref |
