# Supplementary material for: Path2Models: large-scale generation of computational models from biochemical pathway maps
Source: BMC Syst Biol. 2013 Nov 1;7:116. doi: 10.1186/1752-0509-7-116 (PMC4228421; doi:10.1186/1752-0509-7-116)
Supplement: Additional file 2 — Provided as an additional file and through labarchives, DOI:10.6070/H4WH2MX0. [file 1752-0509-7-116-S2.zip › Subliminal Toolbox v2/doc/mcisb-subliminal-lite/overview-summary.html]

Overview


---


|  |  |  |  |  |  |  |  |  |  |
| --- | --- | --- | --- | --- | --- | --- | --- | --- | --- |
| |  |  |  |  |  |  |  | | --- | --- | --- | --- | --- | --- | --- | | **Overview** | Package | Class | **Tree** | **Deprecated** | **Index** | **Help** | | |  |
| PREV   NEXT | **FRAMES**    **NO FRAMES**     **All Classes** |


---

| **Packages** | |
| --- | --- |
| **org.mcisb.subliminal\_lite** |  |
| **org.mcisb.subliminal\_lite.kegg** |  |
| **org.mcisb.subliminal\_lite.merge** |  |
| **org.mcisb.subliminal\_lite.metacyc** |  |
| **org.mcisb.subliminal\_lite.mnxref** |  |
| **org.mcisb.subliminal\_lite.model** |  |
| **org.mcisb.subliminal\_lite.sbml** |  |
| **org.mcisb.subliminal\_lite.xref** |  |

---


|  |  |  |  |  |  |  |  |  |  |
| --- | --- | --- | --- | --- | --- | --- | --- | --- | --- |
| |  |  |  |  |  |  |  | | --- | --- | --- | --- | --- | --- | --- | | **Overview** | Package | Class | **Tree** | **Deprecated** | **Index** | **Help** | | |  |
| PREV   NEXT | **FRAMES**    **NO FRAMES**     **All Classes** |


---
