# Supplementary material for: Path2Models: large-scale generation of computational models from biochemical pathway maps
Source: BMC Syst Biol. 2013 Nov 1;7:116. doi: 10.1186/1752-0509-7-116 (PMC4228421; doi:10.1186/1752-0509-7-116)
Supplement: Additional file 2 — Provided as an additional file and through labarchives, DOI:10.6070/H4WH2MX0. [file 1752-0509-7-116-S2.zip › Subliminal Toolbox v2/doc/mcisb-subliminal-lite/overview-tree.html]

Class Hierarchy


---


|  |  |  |  |  |  |  |  |  |  |
| --- | --- | --- | --- | --- | --- | --- | --- | --- | --- |
| |  |  |  |  |  |  |  | | --- | --- | --- | --- | --- | --- | --- | | **Overview** | Package | Class | **Tree** | **Deprecated** | **Index** | **Help** | | |  |
| PREV   NEXT | **FRAMES**    **NO FRAMES**     **All Classes** |


---


## Hierarchy For All Packages

**Package Hierarchies:**: org.mcisb.subliminal\_lite, org.mcisb.subliminal\_lite.kegg, org.mcisb.subliminal\_lite.merge, org.mcisb.subliminal\_lite.metacyc, org.mcisb.subliminal\_lite.mnxref, org.mcisb.subliminal\_lite.model, org.mcisb.subliminal\_lite.sbml, org.mcisb.subliminal\_lite.xref

---

## Class Hierarchy

- java.lang.Object
  - org.mcisb.subliminal\_lite.xref.**ChebiUtils**- org.mcisb.subliminal\_lite.**Extracter**
      - org.mcisb.subliminal\_lite.model.**BiomassExtracter**- org.mcisb.subliminal\_lite.model.**CobraFormatter**- org.mcisb.subliminal\_lite.kegg.**KeggExtracter**- org.mcisb.subliminal\_lite.metacyc.**MetaCycExtracter**- org.mcisb.subliminal\_lite.model.**TransportExtracter**- org.mcisb.subliminal\_lite.model.**FluxBoundsGenerater**- org.mcisb.subliminal\_lite.model.**GeneAssociationGenerater**- org.mcisb.subliminal\_lite.kegg.**KeggUtils**- org.mcisb.subliminal\_lite.kegg.**KeggUtilsTest**- org.mcisb.subliminal\_lite.metacyc.**MetaCycUtils**- org.mcisb.subliminal\_lite.metacyc.**MetaCycUtilsTest**- org.mcisb.subliminal\_lite.model.**ModelGenerater**- org.mcisb.subliminal\_lite.mnxref.**MxnRefChemUtilsTest**- org.mcisb.subliminal\_lite.mnxref.**MxnRefReactionUtilsTest**- org.mcisb.subliminal\_lite.mnxref.**MxnRefUtils**
                          - org.mcisb.subliminal\_lite.mnxref.**MxnRefChemUtils**- org.mcisb.subliminal\_lite.mnxref.**MxnRefReactionUtils**- org.mcisb.subliminal\_lite.**Path2ModelsReconstructionGenerator**- org.mcisb.subliminal\_lite.sbml.**SbmlFactory**- org.mcisb.subliminal\_lite.merge.**SimpleMerger**- org.mcisb.subliminal\_lite.**SubliminalUtils**- org.mcisb.subliminal\_lite.**SubliminalUtilsTest**- org.mcisb.subliminal\_lite.model.**XmlFormatter**

## Enum Hierarchy

- java.lang.Object
  - java.lang.Enum<E> (implements java.lang.Comparable<T>, java.io.Serializable)
    - org.mcisb.subliminal\_lite.mnxref.**MxnRefUtils.Evidence**

---


|  |  |  |  |  |  |  |  |  |  |
| --- | --- | --- | --- | --- | --- | --- | --- | --- | --- |
| |  |  |  |  |  |  |  | | --- | --- | --- | --- | --- | --- | --- | | **Overview** | Package | Class | **Tree** | **Deprecated** | **Index** | **Help** | | |  |
| PREV   NEXT | **FRAMES**    **NO FRAMES**     **All Classes** |


---
